# When and how should multiple imputation be used for handling missing data in randomised clinical trials – a practical guide with flowcharts

**DOI:** 10.1186/s12874-017-0442-1

**Published:** 2017-12-06

**Authors:** Janus Christian Jakobsen, Christian Gluud, Jørn Wetterslev, Per Winkel

**Affiliations:** 1 The Copenhagen Trial Unit, Centre for Clinical Intervention Research, Rigshospitalet, Copenhagen University Hospital, Copenhagen, Denmark; 2 Department of Cardiology, Holbæk Hospital, Holbæk, Denmark

**Keywords:** Missing data, Randomised clinical trials, Multiple imputation

## Abstract

**Background:**

Missing data may seriously compromise inferences from randomised clinical trials, especially if missing data are not handled appropriately. The potential bias due to missing data depends on the mechanism causing the data to be missing, and the analytical methods applied to amend the missingness. Therefore, the analysis of trial data with missing values requires careful planning and attention.

**Methods:**

The authors had several meetings and discussions considering optimal ways of handling missing data to minimise the bias potential. We also searched PubMed (key words: missing data; randomi*; statistical analysis) and reference lists of known studies for papers (theoretical papers; empirical studies; simulation studies; etc.) on how to deal with missing data when analysing randomised clinical trials.

**Results:**

Handling missing data is an important, yet difficult and complex task when analysing results of randomised clinical trials. We consider how to optimise the handling of missing data during the planning stage of a randomised clinical trial and recommend analytical approaches which may prevent bias caused by unavoidable missing data. We consider the strengths and limitations of using of best-worst and worst-best sensitivity analyses, multiple imputation, and full information maximum likelihood. We also present practical flowcharts on how to deal with missing data and an overview of the steps that always need to be considered during the analysis stage of a trial.

**Conclusions:**

We present a practical guide and flowcharts describing when and how multiple imputation should be used to handle missing data in randomised clinical.

**Electronic supplementary material:**

The online version of this article (10.1186/s12874-017-0442-1) contains supplementary material, which is available to authorized users.

## Background

The key strength of randomised clinical trials is that random allocation of participants results in similar baseline characteristics in the compared groups – if enough participants are randomised [1, 2]. Hence, in a sufficiently large randomised clinical trial the compared treatment groups are expected to be comparable concerning all observed and unobserved prognostic characteristics at baseline [1, 2]. To maintain this baseline comparability of the compared groups, randomised trials are routinely analysed according to the intention-to-treat principle [1]. However, if some participants are lost to follow-up baseline differences between the compared groups in the analysis may compromise the validity of trial results [1]. Missing data may seriously compromise inferences from randomised clinical trials, especially if missingness is not at random and if missing data are not handled appropriately [3, 4]. The potential bias due to missing data depends on the mechanism causing the data to be missing, and the analytical methods applied [4]. Therefore, the analysis of trial data with missing values requires careful planning and attention.

There are three typical mechanisms causing missing data: missing completely at random (MCAR); missing at random (MAR); and missing not at random (MNAR) [3–5]. The mechanism causing missing data may depend neither on observed data nor on the missing data [4, 5]. Then data are said to be missing completely at random (MCAR) [4, 5]. MCAR causes enlarged standard errors due to the reduced sample size, but does not cause bias (‘systematic error’ that is overestimation of benefits and underestimation of harms) [4]. In this situation, the incomplete datasets are representative for the entire dataset [4]. More often the mechanism of missingness may depend on the observed data [4]. If it only depends on the observed data, then the missing data are missing at random (MAR) given the observed data [4]. MAR allows prediction of the missing values based on the participants with complete data [4]. If the mechanism depends on the missing data, and this dependency remains even given the observed data, then data are classified as missing not at random (MNAR) [4, 5]. The MAR and MNAR conditions cannot be distinguished based on the observed data because by definition the missing data are unknown and it can therefore not be assessed if the observed data can predict the unknown data [4, 5].

In the presence of MAR, methods such as multiple imputation or full information direct maximum likelihood may lead to unbiased results. However, the MAR assumption may not always be clinically plausible [4]. Therefore, sensitivity analyses are often needed to assess the potential impact that MNAR may have on the estimated results [3, 6].

Based on group discussions, review of included papers on this topic, and our personal experience in analysing results of randomised clinical trials, we here present a practical guide with flowcharts on how to deal with missing data when analysing results of randomised clinical trials. We divide our presentation into two sections, of which one is concerned with the planning stage of a randomised clinical trial, while the other focuses on analytical approaches which may prevent bias caused by missing data. We describe the most valid methods used to handle MAR data and proper use of sensitivity analyses to handle MNAR data.

## Methods

The author group had several meetings and discussions considering optimal ways of handling missing data to minimise the potential bias. We studied relevant previous studies based on searches of the literature. We searched the reference lists of known studies for papers (theoretical papers; empirical studies; simulation studies; etc.) on how to deal with missing data when analysing randomised clinical trials. We also searched PubMed (last search 14th September 2017) identifying 166 studies using the key words ‘missing data’, ‘randomi*’, and ‘statistical analysis’).

## Results

### The planning stage of a randomised clinical trial

To prevent the occurrence of missing data, a randomised trial must be planned in every detail to reduce the risks of missing data [3, 6]. Before randomisation, the participants’ registration numbers and values of stratification variables should be registered and relevant practical measures ought to be used to limit missingness of key data items. As further steps to prevent missing values we suggest the following three essential components:

1. Before the randomisation begins all statistical analyses should be specified in detail and a statistical analysis plan should be available at a website, registered (for example, at clinicaltrials.gov), or ideally peer-reviewed and published [7]. The statistical analysis plan can either be part of the protocol or a separate document. These steps towards transparency help people declare their preconceived ideas for the statistical analysis, including how to prevent missing data and how to handle missing data [7–10].
2. Key data items should be identified in the statistical analysis plan of the protocol and missingness of these items should be planned to be flagged during data entry, so it is possible during the trial to monitor the extent of the missing data and to intervene and prevent the missingness if possible. Such monitoring and corrective actions need to be described in the data management plan of the trial [7].
3. The procedures necessary to prevent missing key data items should be described in the protocol, and the person(s) responsible for dealing with these problems should be identified so these procedures may be used during the trial period.

Relevant practical measures aiming at limiting missing key data items will vary from trial to trial, and specific recommendations should be tailored for each trial. It must be stressed that limiting the missingness of key data items is crucial and will often be more important than choosing validly between different statistical methods used to deal with missing data.

### The analysis stage of a randomised clinical trial

#### General principles when analysing trial data

The analyses necessitated by the statistical analysis plan may be broken down into a set of regression analyses each including one or more pairwise comparisons of interventions (for example, experimental drug versus placebo). Each regression analysis has a single dependent (outcome) variable (single value regression analysis). When longitudinal data are analysed, a panel of outcomes contains values of the same quantity, but measured at different times relative to the time of the participants’ randomisation, and any exceptions from the pre-planned timing should be noted and discussed. The primary regression analyses should only include as covariates an intervention indicator (for example, experimental drug versus placebo), the protocol specified stratification variables (for example, centre, sex, age), and the baseline value of the dependent variable (if it is a continuous dependent variable) [11, 12]. This implies a considerable simplification of the missing value problem and implies that quite simple and theoretically sound methods may often be applied. Using these principles, we will address the single value regression analysis in the following.

## Methods to handle missing data

When data are ready to be analysed, it should be thoroughly assessed, based on inspection of the data, whether statistical methods ought to be used to handle missing data. Bell et al. aimed to assess the extent and handling of missing data in randomised clinical trials published between July and December 2013 in the BMJ, JAMA, Lancet, and New England Journal of Medicine [13]. 95% of the 77 identified trials reported some missing outcome data. The most commonly used method to handle missing data in the primary analysis was complete case analysis (45%), single imputation (27%), model-based methods (for example, mixed models or generalised estimating equations) (19%), and multiple imputation (8%) [13].

### Complete case analysis

Complete case analysis is statistical analysis based on participates with a complete set of outcome data. Participants with any missing data are excluded from analysis. As described in the introduction, if the missing data are MCAR the complete case analysis will have a reduced statistical power due to the reduced sample size, but the observed data will not be biased [4]. When missing data are not MCAR, the complete case analysis estimate of the intervention effect might be based, i.e., there will often be a risk of overestimation of benefit and underestimation of harm [5, 14–17]. Please see the section ‘Should multiple imputation be used to handle missing data?’ for a more detailed discussion of the potential validity if the complete case analysis is applied.

### Single imputation

When using single imputation, missing values are replaced by a value defined by a certain rule [5]. There are many forms of single imputation, for example, last observation carried forward (a participant’s missing values are replaced by the participant’s last observed value), worst observation carried forward (a participant’s missing values are replaced by the participant’s worst observed value), and simple mean imputation [5]. In simple mean imputation, missing values are replaced by the mean for that variable [5]. Using single imputation often result in an underestimation of the variability because each unobserved value carries the same weight in the analysis as the known, observed values [5]. The validity of single imputation does not depend on whether data are MCAR; single imputation rather depend on specific assumptions that the missing values, for example are identical to the last observed value [5]. These assumptions are often unrealistic and single imputation is therefore often a potentially biased method and should be used with great caution [5, 18, 19].

### Multiple imputation

Multiple imputation has been shown to be a valid general method for handling missing data in randomised clinical trials, and this method is available for most types of data [4, 18–22]. We will in the following sections describe when and how multiple imputation should be used.

### Should multiple imputation be used to handle missing data?

#### Reasons why multiple imputation should not be used to handle missing data

##### Is it valid to ignore missing data?

Analysis of observed data (complete case analysis) ignoring the missing data is a valid solution in three circumstances.

- Complete case analysis may be used as the primary analysis if the proportions of missing data are below approximately 5% (as a rule of thumb) and it is implausible that certain patient groups (for example, the very sick or the very ‘well’ participants) specifically are lost to follow-up in one of the compared groups [23, 24]. In other words, if the potential impact of the missing data is negligible, then the missing data may be ignored in the analysis [23, 24]. Best-worst and worst-best case sensitivity analyses [24, 25] may be used if in doubt: first a ‘best-worst-case’ scenario dataset is generated where it is assumed that all participants lost to follow-up in one group (referred to as group 1) have had a beneficial outcome (for example, had no serious adverse event); and all those with missing outcomes in the other group (group 2) have had a harmful outcome (for example, have had a serious adverse event) [23, 24]. Then a ‘worst-best-case’ scenario dataset is generated where it is assumed that all participants lost to follow-up in group 1 have had a harmful outcome; and that all those lost to follow-up in group 2 have had a beneficial outcome [23, 24]. If continuous outcomes are used, then a ‘beneficial outcome’ might be the group mean plus 2 standard deviations (or 1 standard deviation) of the group mean, and a ‘harmful outcome’ might be the group mean minus 2 standard deviations (or 1 standard deviation) of the group mean [23, 24]. For dichotomised data, these best-worst and worst-best case sensitivity analyses will then show the range of uncertainty due to missing data, and if this range does not give qualitatively contradicting results, then the missing data may be ignored. For continuous data imputation with 2 SD will represent a possible range of uncertainty given 95% of the observed data (if normally distributed).
- If only the dependent variable has missing values and auxiliary variables (variables not included in the regression analysis, but correlated with a variable with missing values and/or related to its missingness) are *not* identified, complete case analysis may be used as the primary analysis and no specific methods ought to be used to handle the missing data [20]. No additional information will be obtained by, for example, using multiple imputation [20] but the standard errors may increase due to the uncertainty introduced by the multiple imputation [20].
- As mentioned above (see Methods to handle missing data), it would also be valid just to perform complete case analysis if it is relatively certain that the data are MCAR (see Introduction). It is relatively rare that it is certain that the data are MCAR. It is possible to test the hypothesis that the data are MCAR with Little’s test [1], but it may be unwise to build on tests that turned out to be insignificant. Hence, if there is reasonable doubt if the data are MCAR, even if Little’s test is insignificant (fail to reject the null hypothesis that data is MCAR), then MCAR should not be assumed.

##### Are the proportions of missing data too large?

If large proportions of data are missing it ought to be considered just to report the results of the complete case analysis and then clearly discuss the resulting interpretative limitations of the trial results. If multiple imputations or other methods are used to handle missing data it might indicate that the results of the trial are confirmative, which they are not if the missingness is considerable. If the proportions of missing data are very large (for example, more than 40%) on important variables, then trial results may only be considered as hypothesis generating results [26]. A rare exception would be if the underlying mechanism behind the missing data can be described as MCAR (see paragraph above).

##### Do the MCAR and the MAR assumption both seem implausible?

If the MAR assumption seems implausible based on the characteristics of the missing data, then trial results will be at risk of biased results due to ‘incomplete outcome data bias’ [27] and no statistical method can with certainty take account of this potential bias [4, 5]. The validity of methods used to handle MNAR data require certain assumptions that cannot be tested based on observed data. Best-worst and worst-best case sensitivity analyses may show the full theoretical range of uncertainty and conclusions ought to be related to this range of uncertainty. The limitations of the analyses should be thoroughly discussed and considered.

##### Is the outcome variable with missing values continuous and is the analytical model complicated (e.g. with interactions)?

In this situation, one may consider using the direct maximum likelihood method to avoid the problems of model compatibility between the analytical model and the multiple imputation model where the former is more general than the latter. In general, direct maximum likelihood methods may be used, but to our knowledge commercially available methods are at present only available for continuous variables.

#### When and how to use multiple imputations

**Fig. 1:**
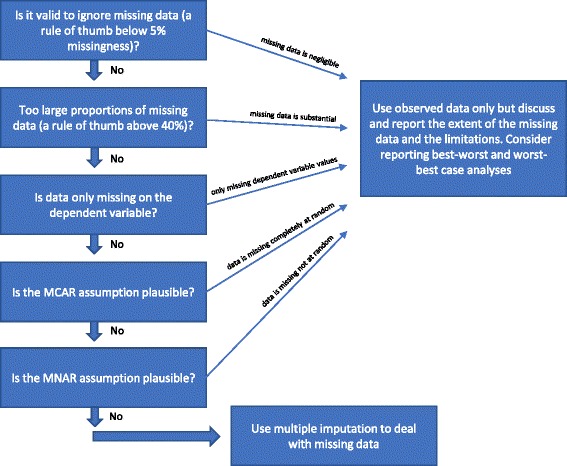
Flowchart: when should multiple imputation be used to handle missing data when analysing results of randomised clinical trials

If none of the ‘Reasons why multiple imputation should not be used to handle missing data’ from above is fulfilled, then multiple imputation could be used. Various procedures have been suggested in the literature over the last several decades to deal with missing data [22]. We have outlined the above-mentioned considerations of statistical methods to handle missing data in Fig. 1.

Multiple imputation originated in the early 1970s, and has gained increasing popularity over the years [22]. Multiple imputation is a simulation-based statistical technique for handling missing data [7]. Multiple imputation consists of three steps:

1. Imputation step. An ‘imputation’ generally represents one set of plausible values for missing data – multiple imputation represents multiple sets of plausible values [7]. When using multiple imputation, missing values are identified and are replaced by a random sample of plausible values imputations (completed datasets). Multiple completed datasets are generated via some chosen imputation model [22]. Five imputed datasets have traditionally been suggested to be sufficient on theoretical grounds, but 50 datasets (or more) seem preferable to reduce sampling variability from the imputation process [4, 21, 22].
2. Completed-data analysis (estimation) step. The desired analysis is performed separately for each dataset that is generated during the imputation step [22]. Hereby, for example, 50 analysis results are constructed.
3. Pooling step. The results obtained from each completed-data analyses are combined into a single multiple-imputation result [22]. There is no need to conduct a weighted meta-analysis as all say 50 analysis results are considered to have the same statistical weight.

It is of great importance that there is either compatibility between the imputation model and the analysis model or the imputation model is more general than the analysis model (for example, that the imputation model includes more independent covariates than the analysis model) [28]. For example, if the analysis model has significant interactions, then the imputation model should include them as well [28], if the analysis model uses a transformed version of a variable then the imputation model should use the same transformation [28], etc.

##### Different types of multiple imputation

Different types of multiple imputation methods exist. We will present them according to their increasing degrees of complexity: 1) single value regression analysis; 2) monotonic imputation; 3) chained equations or the Markov chain Monte Carlo (MCMC) method. We will in the following paragraphs describe these different multiple imputation methods and how to choose between them.

A single variable regression analysis includes a dependent variable and the stratification variables used in the randomisation. The stratification variables often include a centre indicator if the trial is a multi-centre trial and usually one or more adjusting variables with prognostic information which are correlated with the outcome. When using a continuous dependent variable, a baseline value of the dependent variable may also be included. As mentioned in ‘Reasons why statistical methods should not be used to handle missing data’, if only the dependent variable has missing values and auxiliary variables are *not* identified, a complete case analysis should be performed and no specific methods ought to be used to handle the missing data [20]. If auxiliary variables have been identified, a single variable imputation may be performed. If there are significant missingness on the baseline variable of a continuous variable, a complete case analysis may provide biased results [4]. Therefore, in all events, a single variable imputation (with or without auxiliary variables included as appropriate) is conducted if only the baseline variable is missing.

If both the dependent variable and the baseline variable are missing and the missingness is monotone, a monotonic imputation is done. Assume a data matrix where patients are represented by rows and variables by columns. The missingness of such a data matrix is said to be monotone if its columns can be reordered such that for any patient (a) if a value is missing all values to the right of its position are also missing, and (b) if a value is observed all values to the left of this value are also observed [20]. If the missingness is monotone, the method of multiple imputation is also relatively straightforward, even if more than one variable has missing values [20]. In this case it is relatively simple to impute the missing data using sequential regression imputation where the missing values are imputed for each variable at a time [20]. Many statistical packages (for example, STATA) may analyse if the missingness is monotone or not.

**Fig. 2:**
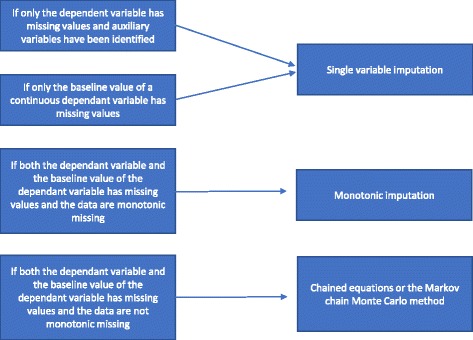
Flowchart of multiple imputation

If missingness is not monotone, a multiple imputation is conducted using the chained equations or the MCMC method. Auxiliary variables are included in the model if they are available. We have summarised how to choose between the different multiple imputation methods in Fig. 2.

### Full information maximum likelihood

Full information maximum likelihood is an alternative method for dealing with missing data [28]. The principle of maximum likelihood estimation is to estimate parameters of the joint distribution of outcome (Y) and covariates (X1,…, X_k_) that, if true, would maximise the probability of observing the values that we in fact observed [28, 29]. If values are missing in a given patient, we can obtain the likelihood by summing the usual likelihood over all possible values of the missing data provided the missing data mechanism is ignorable. This method is referred to as full information maximum likelihood [28, 29].

Full information maximum likelihood has both strengths and limitations compared to multiple imputation.

#### Strengths of full information maximum likelihood compared to multiple imputation

1. It is simpler to implement, i.e. it is not necessary to go through different steps as when using multiple imputation.
2. Unlike multiple imputation, full information maximum likelihood has no potential problems with incompatibility between the imputation model and the analysis model (see ‘Multiple imputation’). The validity of the multiple imputation results will be questionable if there is an incompatibility between the imputation model and the analysis model, or if the imputation model is less general than the analysis model [28].
3. When using multiple imputation, all missing values in each generated dataset (imputation step) are replaced by a random sample of plausible values [22]. Hence, unless ‘a random seed’ is specified, each time a multiple imputation analysis is performed different results will be shown [22]. Analyses when using full information maximum likelihood on the same data set will produce the same results each time the analysis is performed, and the results are therefore not dependent on a random number seed. However, if the random seed value is defined in the statistical analysis plan this problem may be solved.

#### Limitations of full information maximum likelihood compared to multiple imputation

The limitations of using full information maximum likelihood compared to using multiple imputation, is that using full information maximum likelihood is only possible using specially designed software [28]. Designed preliminary software have been developed, but most of these lacks the features of commercially designed statistical software (for example, STATA, SAS, or SPSS). In STATA (using the SEM command) and SAS (using the PROC CALIS command), it is possible to use full information maximum likelihood but only when using continuous dependent (outcome) variables. For logistic regression and Cox regression, the only commercial package that does provide full information maximum likelihood for missing data is Mplus.

A further potential limitation when using full information maximum likelihood is that there may be an underlying assumption of multivariate normality [28]. Nevertheless, violations of the multivariate normality assumption may not be that important so it might be acceptable to include binary independent variables in the analysis [28].

We have in Additional file 1 included a program (SAS) that produces a full toy dataset including several different analyses of these data. Table 1 and Table 2 show the output and how different methods that handle missing data produce different results.

**Table 1:** Estimated regression coefficients and standard errors (SE) of data with no values missing; when values are missing completely at random; when outcome blood pressure (BP) is missing at random; when covariate (baseline BP) is missing at random; and when outcome BP is missing not at random

Type of missingness	Randomised groups	Systolic blood pressure (mmHg) at baseline mean (SE) N	Systolic blood pressure (mmHg) at end of trial mean (SE) N	Parameters		
				Intercept estimate (SE) P	Baseline blood pressure estimate (SE) P	Intervention estimate (SE) P
None	Experimental *N* = 103	181.6 (2.90) *N* = 103	130.8 (3.17) *N* = 103	−2.48 (4.69) *P* = 0.60	1.013 (0.025) *P* < 0.0001	−50.8 (1.48) *P* < 0.0001
	Control *N* = 97	180.9 (2.98) *N* = 97	180.9 (3.13) *N* = 97			
MCAR	Experimental *N* = 103	181.6 (3.64) *N* = 74	131.0 (4.02) *N* = 74	−6.85 (5.22) *P* = 0.19	1.041 (0.028) *P* < 0.0001	−51.2 (1.69) *P* < 0.0001
	Control *N* = 97	181.8 (3.43) *N* = 75	182.3 (3.71) *N* = 75			
MAR (outcome missing)	Experimental *N* = 103	181.6 (2.90) *N* = 103	129.7 (3.97) *N* = 72	−2.75 (5.13) *P* = 0.59	1.015 (0.028) *P* < 0.0001	−51.2 (1.66) *P* < 0.0001
	Control *N* = 97	181.0 (2.98) *N* = 97	180.9 (3.13) *N* = 97			
MAR (baseline missing)	Experimental *N* = 103	181.6 (2.90) *N* = 103	130.8 (3.17) *N* = 103	−5.32 (5.56) *P* = 0.34	1.004 (0.034) *P* < 0.0001	−46.2 (2.22) *P* < 0.0001
	Control *N* = 97	156.2 (2.82) *N* = 36	180.9 (3.13) *N* = 97			
MNAR (outcome missing)	Experimental *N* = 103	181.6 (2.90) *N* = 103	127.8 (3.24) *N* = 95	−8.13 (5.67) *P* = 0.15	1.026 (0.032) *P* < 0.0001	−47.6 (2.12) *P* < 0.0001
	Control *N* = 97	181.0 (2.98) *N* = 97	163.4 (5.37) *N* = 38			

The analyses used in all scenarios were a complete case analysis

**Table 2:** Estimated regression coefficients and standard errors (SE) when no values are missing; when data are missing completely at random; when outcome blood pressure (BP) is missing at random; when covariate (baseline BP) is missing at random; and when outcome BP is missing not at random. When values were missing, multiple imputation as well as the maximum likelihood method were used

Type of missingness	Analysis	Regression coefficients		
		Intercept estimate (standard error (SE)) P	Baseline blood pressure estimate (SE) P	Outcome blood pressure estimate (SE) P
No missing values	Complete case analysis	−2.48 (4.69) 0.60	1.013 (0.025) <0.0001	−50.8 (1.48) *P* < 0.0001
Missing completely at random (MCAR)	Multiple imputation	−6.11 (5.72) *P* = 0.29	1.037 (0.030) *P* < 0.0001	−51.5 (1.78) *P* < 0.0001
	Maximum likelihood	−6.85 (5.17) *P* = 0.18	1.041 (0.028) *P* < 0.0001	−51.2 (1.68) *P* < 0.0001
Missing at random (MAR) (outcome)	Multiple imputation	−2.60 (5.15) *P* = 0.61	1.014 (0.028) *P* < 0.0001	−51.0 (1.70) *P* < 0.0001
	Maximum likelihood	−2.75 (5.08) *P* = 0.59	1.015 (0.027) *P* < 0.0001	−51.2 (1.65) *P* < 0.0001
Missing at random (MAR) (baseline blood pressure)	Multiple imputation	−6.09 (5.37) *P* = 0.26	1.026 (0.029) *P* < 0.0001	−51.1 (2.16) *P* < 0.0001
	Maximum likelihood	−5.49 (5.41) *P* = 0.31	1.026 (0.032) *P* < 0.0001	−50.2 (2.18) *P* < 0.0001
Not missing at random (MNAR) (outcome blood pressure)	Multiple imputation	−8.64 (5.07) *P* = 0.089	1.026 (0.028) *P* < 0.0001	−47.5 (1.99) *P* < 0.0001
	Maximum likelihood	−8.13 (5.61) *P* = 0.15	1.026 (0.032) *P* < 0.0001	−47.6 (2.09) *P* < 0.0001

For comparison the results of an analysis of the data without any values missing is also shown

### Panel values regression analysis

Panel data are usually contained in a so-called wide data file where the first row contains the variable names, and subsequent rows (one for each patient) contain the corresponding values. The outcome is represented by different variables – one for each planned, timed measurement of the outcome. To analyse the data, one must convert the file to a so-called long file with one record per planned outcome measurement, including the outcome value, the time of measurement, and a copy of all other variable values excluding those of the outcome variable. To retain the within-patient correlations between the timed outcome measurements, it is common practice to perform a multiple-imputation of the data file in its wide form followed by an analysis of the resulting file after it has been converted to its long form. Proc mixed (SAS 9.4) may be used for the analysis of continuous outcome values and proc. glimmix (SAS 9.4) for other types of outcome. Because these procedures apply the direct maximum likelihood method on the outcome data, but ignore cases with missing covariate values, the procedures may be used directly when only dependent variable values are missing, and no good auxiliary variables are available. Otherwise, proc. mixed or proc. glimmix (whichever is appropriate) should be used after a multiple-imputation. Clearly, a corresponding approach may be possible using other statistical packages.

### Sensitivity analyses

Sensitivity analyses may be defined as a set of analyses where data are handled in a different way as compared to the primary analysis. Sensitivity analyses may show how assumptions, different from those made in the primary analysis influence the results obtained [3, 6]. Sensitivity analysis ought to be predefined and described in the statistical analysis plan, but additional post hoc sensitivity analyses might be warranted and valid. When the potential influence of missing values is unclear, we recommend the following sensitivity analyses:

1. We have already described the use of best-worst and worst-best case sensitivity analyses to show the range of uncertainty due to missing data (see Assessment of whether methods ought to be used to handle missing data). Our previous description of the best-worst and worst-best case sensitivity analyses was related to missing data on either a dichotomous or a continuous dependent variable, but these sensitivity analyses may also be used when data are missing on stratification variables, baseline values, etc. The potential influence of missing data should be assessed for each variable separately, i.e., there should be one best-worst and one worst-best case scenario for each variable (dependent variable, the outcome indicator, and the stratification variables) with missing data.
2. If it is decided that, for example, multiple imputations should be used, then these results should be the primary result of the given outcome. Each primary regression analysis should always be supplemented by a corresponding observed (or available) case analysis.

### When mixed-effect methods are used

Using a multi-centre trial design will often be necessary to recruit a sufficient number of trial participants within a reasonable time frame [30]. A multi-centre trial design also provides a better basis for the subsequent generalisation of its findings [30]. It has been shown that the most commonly used analysis methods in randomised clinical trials perform well with a small number of centres (analysing binary dependent outcomes) [31]. With a relatively large number of centres (50 or more), it is often optimal to use ‘centre’ as a random effect and to use mixed effect analysis methods. It will often also be valid to use mixed-effect analysis methods when analysing longitudinal data [32]. It might in some circumstances be valid to include the ‘random effect’ covariate (for example ‘centre’) as a fixed-effect covariate during the imputation step and then use mixed model analysis or generalised estimating equations (GEE) during the analysis step [29, 33]. However, the application of a mixed-effects model (with, for example, ‘centre’ as a random effect) implies that the multi-layered structure of the data must be taken into consideration when modelling the multiple imputation. Now, commercial software is not directly available to do so. However, one may use the REALCOME package which may be interfaced with STATA [22]. The interface exports the data with missing values from STATA to REALCOM where the imputation is done taking the multilevel nature of the data into account and using a MCMC method which includes continuous variables and by using a latent normal model also allows a proper handling of discrete data [22]. The imputed datasets may then be analysed using the STATA ‘mi estimate:’ command which may be combined with the ‘mixed’ statement (for a continuous outcome) or the ‘meqrlogit’ statement for binary or ordinal outcome in STATA [22]. In the analysis of panel data, however, one may easily find oneself confronted with a situation where data include three or more levels, for example, measurements within the same patient (level-1), patients within centres (level-2), and centres (level-3) [22]. Not to get involved with a rather complicated model which may lead to lack of convergence or unstable standard errors and for which commercial software is not available, we would recommend either treating the centre effect as fixed (directly or following the merging of small centres into one or more appropriately sized centres, using a procedure that must be prescribed in the statistical analysis plan) or exclude centre as a covariate. If randomisation has been stratified by centre, the latter approach will lead to an upward bias of the standard errors resulting in a somewhat conservative test procedure [12].

## Discussion

Missing data will always be a limitation when interpreting trial results; even if the data are MCAR, the missing data will result in loss of statistical power. These limitations due to missing data should always be thoroughly considered and discussed by the trialists. As always, prevention is better than cure. To mount professional prevention, trials need to be focused and pragmatic. Trial results based on data with missing values should always be interpreted with caution. It is not possible to differentiate between MAR and MNAR so the validity of the underlying assumptions behind, for example, multiple imputation may always be questioned, and when the data are MNAR, no methods exist to handle missing data appropriately. However, the best-worst and worst-best case analyses will for dichotomised data always show the widest possible range of uncertainty and for continuous data a possible range of uncertainty given 95% of the normally distributed observed data. The primary conclusion on intervention effects should often be related to the this shown range of uncertainty.

Handling missing data validly is an important, yet difficult and complex, task. We have presented practical flowcharts on how to deal with missing data when analysing results of randomised clinical trials. It is beyond the scope of this paper to describe how to deal with the multiple and often very complex statistical issues when, for example, using multiple imputation. It is often advisable to consult knowledgeable persons with statistical expertise when analysing trial results, and this paper does not in any way change this need. However, we have presented a practical guide and an overview of the steps that always need to be considered during the analysis stage of a trial.

## Conclusion

We present a practical guide and flowcharts describing when and how multiple imputation should be used to handle missing data in randomised clinical trials.

## Additional file

Additional file 1: Program (SAS) that produces a full toy dataset including several different analyses of these data. (DOCX 16 kb)

## Abbreviations

MAR: Missing at random
MCAR: Missing completely at random
MCMC: Markov chain Monte Carlo
MNAR: Missing not at random
