# Supplementary material for: When and how should multiple imputation be used for handling missing data in randomised clinical trials – a practical guide with flowcharts
Source: BMC Med Res Methodol. 2017 Dec 6;17:162. doi: 10.1186/s12874-017-0442-1 (PMC5717805; doi:10.1186/s12874-017-0442-1)
Supplement: Additional file 1: — Program (SAS) that produces a full toy dataset including several different analyses of these data. (DOCX 16 kb) [file 12874_2017_442_MOESM1_ESM.docx]

/*DATA SET IS CONSTRUCTED */

/*Ranuni (seed) is a function which generates a pseudorandom number in the interval [0,1].

Rannor (seed) generates a random draw from the standardized normal distribution. Seed is an arbitrary integer. */

**data** test;

do J = **1** to **200**;

PTID=j;

if ranuni(**1**) GT **0.5** then intervention = **1**; else intervention = **0**;/*patients are randomised*/

BP0 = **180** + **30***rannor(**1**); /*baseline pressure is constructed */

if intervention EQ **1** then BPoutcome = BP0 -(**50** + rannor(**2**)***10**); /*effect of intervention constructed*/

else BPoutcome = BP0 - rannor(**3**)***10**; /* In all events outcome is correlated with baseline value */

drop J;

output;

end;

**run**;

/* Various types of missingness are now created */

**data** test0; /* 20% drop outs. MCAR */

set test;

if ranuni(**7**) GT **0.80** then do;

BP0 = **.**; /* 20% missing completely at random */

BPoutcome =**.**;

end;

**run**;

**data** test1; /*Outcome is missing. MAR */

set test;

if intervention EQ **1** AND ranuni(**1**) GT **0.65** then BPoutcome = **.**; /* 35% outcomes missing in experimental arm */

**run**;**quit**;

**data** test2;/*covariat (i.e. baseline pressure) is missing MAR*/

set test;

if BPoutcome GE **170** AND ranuni(**1**) GT **0.01** AND intervention EQ **0** then BP0 = **.**; /* high outcome BP 99% missing baseline BP in control group*/

**run**;**quit**;

**data** test3; /*Outcome is missing NMAR*/

set test;

cut = **0.65**;

if intervention EQ **0** then cut = **0.20**;/* The subjective effect of the high BP is higher in the experimental arm */

if ranuni(**1**) GT cut AND BPoutcome GT **150** then BPoutcome = **.**; /* High outcome BP missing because it is high */

**run**;**quit**;

/* COMPLETE CASE ANALYSES */

/* regression analysis of test data without missing values */

**proc** **reg** data=test;

model BPoutcome =BP0 intervention;

**run**;

**proc** **means** data = test N mean STD STDERR MIN MAX MAXDEC=**2**;

CLASS INTERVENTION;

VAR BP0 BPoutcome;

**RUN**;

/* MCAR data */

**proc** **reg** data=test0;

model BPoutcome =BP0 intervention;

**run**;

**proc** **means** data = test0 N mean STD STDERR MIN MAX MAXDEC=**2**;

CLASS INTERVENTION;

VAR BP0 BPoutcome;

**RUN**;

/*complete case analysis outcome MAR */

**proc** **reg** data=test1;

model BPoutcome =BP0 intervention;

**run**;

**proc** **means** data = test1 N mean STD STDERR MIN MAX MAXDEC=**2**;

CLASS INTERVENTION;

VAR BP0 BPoutcome;

**RUN**;

/* Complete case analysis covariate has missing values (MAR) */

**proc** **reg** data=test2;

model BPoutcome =BP0 intervention;

**run**;

**proc** **means** data = test2 N mean STD STDERR MIN MAX MAXDEC=**2**;

CLASS INTERVENTION;

VAR BP0 BPoutcome;

**RUN**;

/*Complete case analysis of NMAR data */

**proc** **reg** data=test3;

model BPoutcome = BP0 intervention;

**run**;

**proc** **means** data = test3 N mean STD STDERR MIN MAX MAXDEC=**2**;

CLASS INTERVENTION;

VAR BP0 BPoutcome;

**RUN**;

/***************************************************************************************************/

/* MI AND ML ANALYSES */

/* test0 MCAR*/

**proc** **mi** data=test0 out=test0imp nimpute=**15** seed=**5368**;

var BPoutcome BP0 intervention;

**run**;

**proc** **reg** data=test0imp outest=a covout;

model BPoutcome = BP0 intervention;

by _imputation_;

**run**;

**proc** **mianalyze** data = a;

modeleffects intercept BP0 intervention;

**run**;

**proc** **calis** data=test0 method=fiml;

PATH BPoutcome <- BP0 intervention;

**run**;

/* test1 MAR only outcome is missing */

**proc** **mi** data=test1 out=test1imp nimpute=**15** seed=**5368**;

var BPoutcome BP0 intervention;

**run**;

**proc** **reg** data=test1imp outest=a covout;

model BPoutcome = BP0 intervention;

by _imputation_;

**run**;

**proc** **mianalyze** data = a;

modeleffects intercept BP0 intervention;

**run**;

**proc** **calis** data=test1 method=fiml;

PATH BPoutcome <- BP0 intervention;

**run**;

/* test2 MAR only baseline values are missing */

**proc** **mi** data=test2 out=test2imp nimpute=**15** seed=**5368**;

var BPoutcome BP0 intervention;

**run**;

**proc** **reg** data=test2imp outest=a covout;

model BPoutcome = BP0 intervention;

by _imputation_;

**run**;

**proc** **mianalyze** data = a;

modeleffects intercept BP0 intervention;

**run**;

**proc** **calis** data=test2 method=fiml;

PATH BPoutcome <- BP0 intervention;

**run**;

/* test3 NMAR */

**proc** **mi** data=test3 out=test3imp nimpute=**15** seed=**5368**;

var BPoutcome BP0 intervention;

**run**;

**proc** **reg** data=test3imp outest=a covout;

model BPoutcome = BP0 intervention;

by _imputation_;

**run**;

**proc** **mianalyze** data = a;

modeleffects intercept BP0 intervention;

**run**;

**proc** **calis** data=test3 method=fiml;

PATH BPoutcome <- BP0 intervention;

**run**;

/* FINISHED */
